# Clinical Profile, Severity Markers, and Outcomes of Hospitalized Patients With Scrub Typhus in North India: A Prospective Observational Study

**DOI:** 10.7759/cureus.114289

**Published:** 2026-08-10

**Authors:** Ashish Jangid, Shobhit Agarwal, Dilip Chaurasiya, Shalini Singh, Mohammad Shafat Siddiqui, Neeraj Singh, O. P. Tiwari, Akash Marade, Prashant Pandey, Abhijeet Tripathi

**Affiliations:** 1 General Medicine, Heritage Institute of Medical Sciences, Varanasi, IND; 2 Microbiology, Heritage Institute of Medical Sciences, Varanasi, IND; 3 Internal Medicine, Heritage Institute of Medical Sciences, Varanasi, IND; 4 Pathology, Heritage Institute of Medical Sciences, Varanasi, IND

**Keywords:** aki, ards, hospitalized patients, icu, mods, mortality, scrub typhus

## Abstract

Scrub typhus is an important cause of acute febrile illness in India, with often devastating complications in hospitalized patients. These complications contribute to morbidity and mortality if the condition is not diagnosed early and treated. A prospective observational study was conducted in the Department of General Medicine at a tertiary care teaching hospital in North India from June 2024 to May 2025 and included 35 patients with confirmed scrub typhus. Laboratory parameters, complications, and outcomes were analyzed. Data were analyzed statistically using IBM SPSS Statistics version 25.0. The chi-square or Fisher's exact test was used to test associations with categorical variables. The mean age of patients was 45 ± 16 years, with 20 of 35 patients (57.1%) being male. All 35 patients (100%) had fever, while 24 (68.6%) had myalgia and 23 (65.7%) had headache as the main presenting complaints. An eschar was identified in 10 (28.6%) patients. Major complications included acute kidney injury (AKI) in 10 (28.6%) patients, acute respiratory distress syndrome (ARDS) in 7 (20.0%), and multiple organ dysfunction syndrome (MODS) in 11 (31.4%). Thirteen (37.1%) patients required ICU admission, and 4 (11.4%) died during hospitalization. There was a significant association of MODS, ARDS, and AKI with mortality (*P* < 0.001, *P* < 0.01, and *P* < 0.05, respectively). In hospitalized cases, scrub typhus was associated with significant complications. Morbidity and mortality can be minimized with early clinical suspicion, prompt diagnosis, and timely use of doxycycline-based therapy.

## Introduction

Scrub typhus is a zoonotic disease caused by Orientia tsutsugamushi, which is an important cause of acute undifferentiated febrile illness in tropical and subtropical regions and has been on the rise in the last few years in the endemic *Tsutsugamushi triangle*, including India, where the risk of exposure is increased in rural and semi-urban areas [1-3].

There is a wide spectrum of clinical features, ranging from a mild febrile disease to a severe and potentially fatal illness, and the eschar is more often observed but not always in scrub typhus [4,5]. In severe cases, the infection may cause involvement of several organ systems, including acute kidney injury (AKI), acute respiratory distress syndrome (ARDS), hepatitis, and multiple organ dysfunction syndrome (MODS), which are associated with higher morbidity and mortality [6-8].

One of the most difficult things to manage is the nonspecific clinical presentation of scrub typhus, which mimics other endemic febrile illnesses, including dengue, malaria, and leptospirosis [1,4]. This overlap is often followed by delayed diagnosis and delayed treatment [9,10]. Severity of disease is closely related to the time to therapy, and patients who require hospitalization are usually at the more severe end of the disease spectrum and may require more advanced supportive care.

The incidence of scrub typhus has been rising in India; however, studies specifically evaluating hospitalized patients with severe scrub typhus in North India remain limited. Most published studies have included mixed outpatient and inpatient populations, making it difficult to identify clinical characteristics and predictors of severe disease among hospitalized patients alone. Because hospitalized patients represent the more severe end of the disease spectrum and frequently develop organ dysfunction requiring intensive care, studying this subgroup separately may improve early recognition of severe illness and guide timely management. Therefore, the present study was undertaken to evaluate the clinical profile, laboratory findings, complications, and outcomes of hospitalized patients with scrub typhus at a tertiary care center in North India.

## Materials and methods

This study was designed as a prospective observational study in a tertiary care teaching hospital in North India from June 2024 to May 2025. Only hospitalized patients were included, and the study was conducted in the Department of General Medicine. Thirty-five eligible hospitalized patients with laboratory-confirmed scrub typhus who were admitted consecutively during the study period and fulfilled the inclusion criteria were enrolled. All consecutive eligible patients admitted during the study period who met the inclusion criteria were included in the study.

Patients aged 18 years or older who were hospitalized with laboratory-confirmed scrub typhus based on a positive immunoglobulin M (IgM) enzyme-linked immunosorbent assay (ELISA) test were included in the study. Patients with missing baseline clinical records, incomplete laboratory investigations required for analysis, missing hospital outcome data, or laboratory-confirmed co-infections such as dengue, malaria, or leptospirosis were excluded because these infections have overlapping clinical features with scrub typhus. Other bacterial infections were not routinely evaluated in all patients and were therefore not considered predefined exclusion criteria.

At the time of hospital admission, a detailed clinical history, including demographic characteristics, presenting symptoms, and underlying comorbid illnesses (including diabetes mellitus, hypertension, chronic kidney disease, chronic liver disease, ischemic heart disease, chronic obstructive pulmonary disease, and other significant chronic medical conditions), was obtained, followed by a comprehensive physical examination that included assessment for the presence of an eschar and recording of admission vital signs. Laboratory investigations performed at admission included complete blood count, liver function tests (aspartate aminotransferase (AST), alanine aminotransferase (ALT), and total bilirubin), renal function tests (serum creatinine), serum sodium, serum albumin, and C-reactive protein (CRP). The laboratory values presented in the study represent the baseline investigations obtained at hospital admission before initiation of definitive treatment. Patients were prospectively followed throughout their hospital stay to document complications present at admission as well as those that developed during hospitalization, along with intensive care unit (ICU) admission, requirement for mechanical ventilation, treatment received, and final hospital outcome (recovery or death). All clinical assessments and investigations were performed as part of routine patient care, and no additional diagnostic procedures or interventions were undertaken specifically for the study.

AKI was defined according to the Kidney Disease: Improving Global Outcomes (KDIGO) Clinical Practice Guideline [11]. ARDS was defined according to the Berlin Definition [12]. MODS was defined as dysfunction involving two or more organ systems [13].

Data were entered into Microsoft Excel and analyzed using IBM SPSS Statistics version 25.0 (IBM Corp., Armonk, NY). Continuous variables are presented as mean ± standard deviation, whereas categorical variables are presented as frequencies and percentages. Pearson's chi-square test or Fisher's exact test, as appropriate, was used to compare categorical variables. Continuous variables were compared using the independent-samples t-test. Test statistics (χ² or *t*) with corresponding *P*-values are reported. A *P*-value < 0.05 was considered statistically significant.

## Results

Thirty-five patients with confirmed scrub typhus who were hospitalized in the study area were included in the study. The mean age of the patients was 45 ± 16 years, and 20 (57.1%) were male. All patients (35, 100%) had fever. The mean duration from symptom onset to hospital presentation was 6.1 ± 2.3 days. Baseline comorbid illnesses were present in 16 (45.7%) patients, with hypertension (10, 28.6%) and diabetes mellitus (9, 25.7%) being the most common, followed by chronic kidney disease (6, 17.1%), chronic liver disease (5, 14.3%), ischemic heart disease (3, 8.6%), and chronic obstructive pulmonary disease (2, 5.7%). Some patients had more than one comorbid illness.

Myalgia was present in 24 patients (68.6%) and headache in 23 patients (65.7%), making them the most frequently observed presenting features, followed by vomiting in 14 patients (40.0%) and cough in 14 patients (40.0%). Altered sensorium was present in 5 patients (14.3%), while breathlessness occurred in 8 patients (22.9%). Among the 10 patients (28.6%) with an eschar, the most common sites were the axilla (3, 30%), groin (2, 20%), abdomen (2, 20%), inframammary region (2, 20%), and neck (1, 10%). Post-diagnostic treatment for all patients was doxycycline-based, and supportive treatment was provided based on clinical status. The baseline demographic and clinical characteristics of the study population are summarized in Table 1.

**Table 1:** Baseline clinical characteristics. Patients with ≥1 comorbidity represent the number of patients with one or more pre-existing comorbid illnesses. Individual comorbidities are listed in the table; patients may have had more than one comorbidity; therefore, percentages do not total 100%.

Variable	*n* (%)/Mean ± SD
Total patients	35
Mean age (years)	45 ± 16
Male	20 (57.1)
Fever	35 (100.0)
Myalgia	24 (68.6)
Headache	23 (65.7)
Vomiting	14 (40.0)
Cough	14 (40.0)
Breathlessness	8 (22.9)
Altered sensorium	5 (14.3)
Eschar	10 (28.6)
Comorbidities (≥1 comorbidity)	16 (45.7)
Hypertension	10 (28.6)
Diabetes mellitus	9 (25.7)
Chronic kidney disease	6 (17.1)
Chronic liver disease	5 (14.3)
Ischemic heart disease	3 (8.6)
Chronic obstructive pulmonary disease	2 (5.7)

Baseline laboratory evaluation at hospital admission revealed thrombocytopenia in the majority of patients, with a mean platelet count of approximately 75,000/mm³. Liver enzyme elevation was a consistent finding, with AST levels higher than ALT in most patients. Elevated inflammatory markers and mild hyponatremia were also observed (Table 2).

**Table 2:** Laboratory parameters. SD, standard deviation; AST, aspartate aminotransferase; ALT, alanine aminotransferase; CRP, C-reactive protein

Parameter	Mean ± SD
Hemoglobin (g/dL)	10.5 ± 2.0
Total leukocyte count (/mm³)	9,500 ± 3,000
Platelets (/mm³)	75,000 ± 35,000
AST (IU/L)	180 ± 90
ALT (IU/L)	140 ± 70
Total bilirubin (mg/dL)	1.8 ± 0.9
Creatinine (mg/dL)	1.6 ± 0.8
Sodium (mEq/L)	132 ± 4
CRP (mg/L)	65 ± 40
Albumin (g/dL)	3.1 ± 0.5

Complications present at hospital admission or developing during hospitalization were observed in a significant proportion of patients and are summarized in Table 3. AKI was present in 10 patients (28.6%), ARDS developed in 7 patients (20.0%), and MODS occurred in 11 patients (31.4%). Thirteen patients (37.1%) required ICU admission, and 8 patients (22.9%) required mechanical ventilation.

**Table 3:** Complications and outcomes. AKI, acute kidney injury; ARDS, acute respiratory distress syndrome; MODS, multiple organ dysfunction syndrome; ICU, intensive care unit

Variable	*n* (%)
AKI	10 (28%)
ARDS	7 (20%)
MODS	11 (31%)
ICU admission	13 (37%)
Mechanical ventilation	8 (23%)
Recovered	31 (88.6%)
Mortality	4 (11.4%)

On comparative analysis, severe disease was significantly associated with poor outcomes. The association between clinical variables and mortality is presented in Table 4. All patients who died had evidence of MODS and required ICU admission. Pearson's chi-square analysis demonstrated a significant association between MODS and mortality (χ² = 9.85, *P* = 0.002). Significant associations were also observed for ARDS (χ² = 8.54, *P* = 0.003), AKI (χ² = 11.29, *P* < 0.001), and ICU admission (χ² = 7.64, *P* = 0.006).

**Table 4:** Association of clinical variables with mortality. Associations between categorical variables and mortality were analyzed using Pearson's chi-square test or Fisher's exact test, as appropriate. Percentages for recovered patients are calculated using *n* = 31 as the denominator, and percentages for deaths are calculated using *n* = 4 as the denominator. MODS, multiple organ dysfunction syndrome; ARDS, acute respiratory distress syndrome; AKI, acute kidney injury; ICU, intensive care unit

Variable	Recovered (*n *= 31), *n* (%)	Death (*n* = 4), *n* (%)	*P*-value
MODS	7 (22.6%)	4 (100.0%)	<0.001
ARDS	4 (12.9%)	3 (75.0%)	<0.01
AKI	6 (19.4%)	4 (100.0%)	<0.05
ICU admission	9 (29.0%)	4 (100.0%)	<0.01

Independent-samples t-test demonstrated significantly lower platelet counts among ICU patients compared with non-ICU patients (*t* = -4.56, *P* < 0.001). ICU patients also had significantly higher CRP levels (*t* = 3.73, *P* = 0.002) and serum creatinine concentrations (*t* = 3.32, *P* = 0.004), indicating greater disease severity.

A comparative analysis between ICU and non-ICU patients is shown in Table 5.

**Table 5:** Comparison between ICU and non-ICU patients. Continuous variables were compared using the independent-samples t-test, whereas categorical variables were analyzed using Pearson's chi-square test or Fisher's exact test, as appropriate. CRP, C-reactive protein; MODS, multiple organ dysfunction syndrome; ARDS, acute respiratory distress syndrome

Parameter	ICU patients (*n* = 13)	Non-ICU patients (*n* = 22)	*P*-value
Mean platelet count (/mm³)	52,000 ± 18,000	89,000 ± 30,000	<0.05
Mean CRP (mg/L)	92 ± 38	48 ± 25	<0.05
Mean creatinine (mg/dL)	2.1 ± 0.9	1.2 ± 0.5	<0.05
MODS	9 (69%)	2 (9%)	<0.001
ARDS	6 (46%)	1 (4%)	<0.01
Mechanical ventilation	8 (61%)	0	<0.001
Mortality	4 (31%)	0	<0.01

ICU patients demonstrated significantly lower platelet counts and higher inflammatory markers compared to non-ICU patients. Organ dysfunction, mechanical ventilation requirement, and mortality were significantly more frequent among ICU patients.

These findings suggest that thrombocytopenia, elevated inflammatory markers, and organ dysfunction may serve as markers of severe disease among hospitalized patients with scrub typhus.

## Discussion

The present study aimed to analyze the clinical picture, laboratory findings, and outcomes of hospitalized cases of scrub typhus at the more severe end of the spectrum in a tertiary care center in North India. Fever was the presenting symptom in all patients (35/35, 100%), while myalgia (24/35, 68.6%) and headache (23/35, 65.7%) were the most frequent accompanying complaints. The average duration from symptom onset to hospital presentation in our cohort was approximately six days, which may have contributed to the high frequency of organ dysfunction observed. These observations are consistent with reports from other endemic regions, where fever is the predominant manifestation and constitutional symptoms such as myalgia and headache are commonly encountered during the early phase of illness [4,5,9,10,14,15].

An eschar was identified in 10 of 35 patients (28.6%), which falls within the range reported in previous Indian studies. Most eschars were located at covered body sites, highlighting the importance of a meticulous physical examination, particularly of concealed areas, in patients presenting with acute febrile illness in endemic regions. The frequency of eschar detection varies considerably across different populations and may be influenced by factors such as skin pigmentation, meticulousness of physical examination, anatomical location of the lesion, and geographical variation in circulating strains of Orientia tsutsugamushi. Therefore, although the presence of an eschar is highly suggestive of scrub typhus, its absence should not exclude the diagnosis [4,5,10,15,16].

The laboratory profile of our patients was characterized by thrombocytopenia, elevated hepatic transaminases with a predominance of AST over ALT, increased inflammatory markers, and mild hyponatremia. These abnormalities have been consistently reported in previous studies and reflect the systemic inflammatory response and widespread endothelial injury associated with scrub typhus infection. Similar laboratory patterns have also been associated with increased disease severity and a higher likelihood of organ dysfunction in hospitalized patients [5,7,8,10,15,16,17].

In this study, AKI was observed in 10 of 35 patients (28.6%), ARDS in 7 (20.0%), and MODS in 11 (31.4%). Additionally, 13 patients (37.1%) required ICU admission and 8 (22.9%) required mechanical ventilation. These findings indicate that hospitalized patients with scrub typhus frequently present with severe systemic involvement. Similar rates of organ dysfunction have been described in previous studies, particularly among patients referred to tertiary care centers, where delayed presentation and advanced disease are common [5,6,7,8,14,15,16].

The development of severe scrub typhus is primarily attributed to widespread endothelial infection and vasculitis caused by O. tsutsugamushi. Endothelial injury results in increased vascular permeability, tissue hypoperfusion, and activation of inflammatory pathways, ultimately leading to complications such as AKI, ARDS, and MODS. The thrombocytopenia and elevated inflammatory markers observed in our patients further support the presence of a systemic inflammatory response associated with severe infection [1,7,8,14,18].

All four deaths in our study occurred in patients with severe disease requiring intensive care support. Mortality showed a significant association with the presence of MODS, ARDS, and AKI. Similar observations have been reported in previous studies, which identified delayed diagnosis, respiratory failure, and multiorgan involvement as major predictors of adverse outcomes in scrub typhus [5,7,9,14,16,17]. These findings emphasize the importance of early recognition and timely initiation of appropriate antimicrobial therapy to reduce morbidity and mortality.

Underlying comorbid illnesses such as diabetes mellitus, hypertension, chronic kidney disease, chronic liver disease, ischemic heart disease, and chronic obstructive pulmonary disease may contribute to disease severity by reducing physiological reserve and increasing susceptibility to organ dysfunction. In our cohort, nearly half of the hospitalized patients had at least one pre-existing comorbidity, highlighting the importance of documenting baseline medical conditions during the initial clinical assessment. Although the present study was not powered to evaluate the independent effect of individual comorbidities on outcomes, future larger studies should further investigate their prognostic significance.

On statistical analysis, MODS had a highly significant association with mortality (p < 0.001) and so did ARDS and AKI [6,7]. These results corroborate previous work that has shown that organ dysfunction and involvement of the respiratory system are strong predictors of mortality. In addition, platelet values were significantly lower in patients admitted to the ICU, indicating that platelet count could be a marker of severity in patients admitted to the ICU [8].

The findings of this study have important clinical implications for physicians practicing in endemic regions. Early identification of warning features such as thrombocytopenia, elevated transaminases, hyponatremia, respiratory distress, and evidence of organ dysfunction may facilitate prompt recognition of severe disease and timely escalation of care. Since delayed initiation of appropriate antimicrobial therapy has been associated with poorer outcomes, maintaining a high index of clinical suspicion and starting doxycycline early in suspected cases may help reduce complications and mortality [1,7,10,16,18].

This study has several limitations. First, it was conducted at a single tertiary care center with a relatively small sample size, which may limit the generalizability of the findings. Second, only hospitalized patients were included, introducing the possibility of referral and selection bias toward more severe disease. Third, molecular confirmation by polymerase chain reaction (PCR) was not available for all patients. Finally, long-term follow-up after hospital discharge was not performed, preventing assessment of late outcomes.

## Conclusions

Hospitalized patients with scrub typhus commonly developed serious complications, particularly AKI, ARDS, and MODS, which were significantly associated with adverse hospital outcomes, including mortality. Patients requiring intensive care also had lower platelet counts and higher inflammatory markers, suggesting their potential role as indicators of severe disease. These findings highlight the importance of maintaining a high index of suspicion, recognizing organ dysfunction early, and initiating appropriate antimicrobial therapy without delay in hospitalized patients. Further multicenter studies with larger sample sizes are needed to validate these findings and better define predictors of poor outcomes.
